# Optical generation of strong-field terahertz radiation and its application in nonlinear terahertz metasurfaces

**DOI:** 10.1515/nanoph-2021-0714

**Published:** 2022-01-31

**Authors:** Zhenzhe Ma, Peiyan Li, Sai Chen, Xiaojun Wu

**Affiliations:** School of Electronic and Information Engineering, Beihang University, Beijing 100191, China

**Keywords:** extreme THz, lithium niobate, local field enhancement, nonlinear metasurface, strong-field THz radiation, tilted pulse front

## Abstract

Extremely nonlinear terahertz (THz)-matter interactions and applications have positioned themselves as the next frontier in quantum information, nonlinear optics, and particle acceleration. However, the absence of free-space highly intense THz sources and the diffraction limit, which prevents THz waves from being concentrated to the nanoscale scale, are inhibiting the growth of extreme THz. To address this difficulty, suitably extremely concentrated THz sources are being produced, while (non-)resonant artificial metastructures are being widely used to enhance local fields, resulting in deep-subwavelength (<*λ*/10^3^) confinement of highly enhanced THz fields in micro-/nano-gaps. We discuss solid-state stable sources of intense THz radiation generated by femtosecond lasers in this Review, with a special emphasis on the lithium niobate-based tilted pulse front approach and the nonlinear THz metasurfaces allowed by it. Finally, we forecast the field’s future directions in extreme THz research.

## 1 Introduction

**Figure 1:**
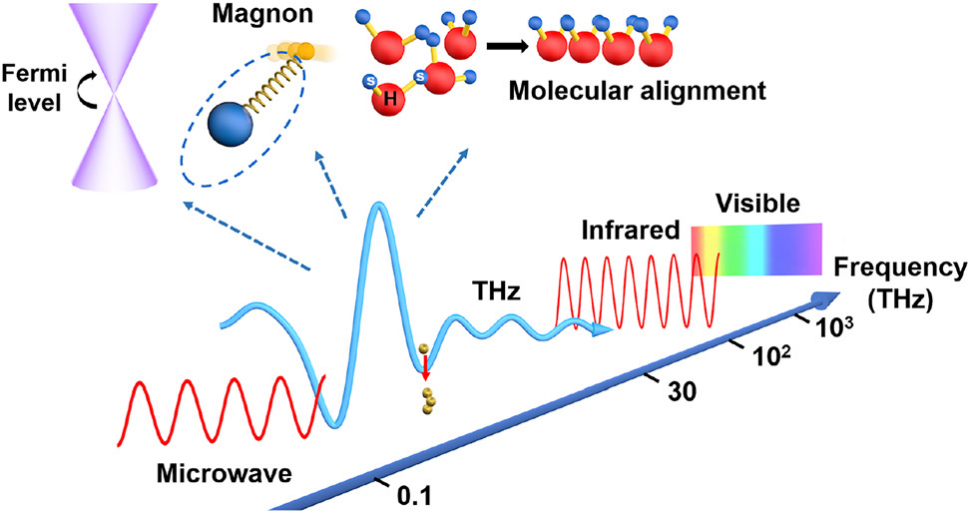
The location of THz frequency range in the electromagnetic spectrum is between microwave and infrared light. THz photon energy is around the Fermi level and its frequency corresponds to some magnon, phonon, or molecular vibration and rotation energy level.

Terahertz (THz) radiation (0.1–30 THz), as indicated in Figure 1, is between the microwave and infrared electromagnetic frequency bands, with photon energies close to the Fermi level and peak electric and magnetic field intensities around or above MV/cm and Tesla, respectively [1]. Additionally, its picosecond/sub-picosecond time resolution enables a wide variety of ultrafast spectroscopic and imaging applications. As a result, it can function as a unique cold light source, revealing a new unexplored realm of the fascinating interaction of light and matter [2–25].

THz frequencies correspond to the intrinsic phonon and magnon vibrations of a large number of strongly correlated systems. Thus, intense THz fields at a certain frequency can stimulate lattice resonance coherently and resonantly, thereby inducing novel electronic structures, discovering new physics, and obtaining new states. It has spawned a new field of research known as lightwave quantum electronics, which is geared toward quantum information processing applications. Additionally, THz pulses can align molecule orientation and hence regulate a large number of catalytic events in chemical engineering [26–29]. THz pulses with a strong field can flip electron spin and enable nonlinear spin control, laying the groundwork for future ultrafast spintronic devices [21, 30], [31], [32], [33], [34], [35], [36]. When combined with scanning probe technology, strong-field THz pulses can generate a tunneling current at the tip of the scanning tunneling microscope (STM), overcoming the diffraction limit of the THz lightwave and providing a new potent instrument for the state management of novel nanoscale materials [6, 37], [38], [39], [40], [41]. Strong-field THz pulses have the ability to accelerate, compress, and manipulate electron micro-bunches in several dimensions, which is predicted to result in the development of table-top miniaturized THz accelerators for use in tiny attosecond X-ray sources [42–53]. Besides, intense THz has been used to examine the biological effects of THz fields in biological applications [54–56].

**Figure 2:**
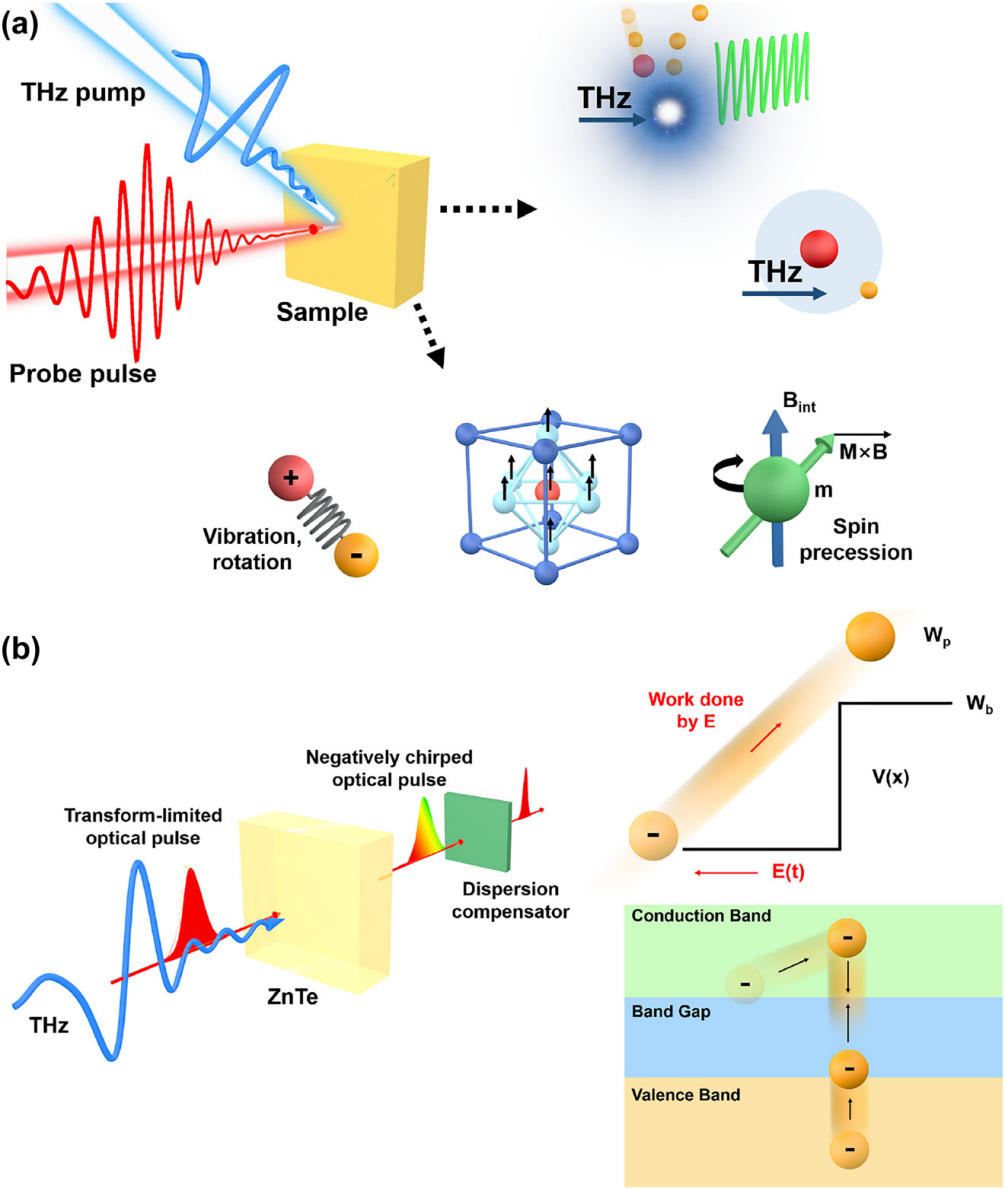
The interactions between strong THz waves and materials. (a) Resonant (such as molecular rotation and crystal lattice vibrations, crystal lattice vibrations, coupling to ionic, electronic, or spin degrees of freedom, etc.) and (b) non-resonant (such as field ionization and impact ionization) interaction between strong THz waves and materials. Reprinted with permission from Kampfrath et al. [21] ©Springer Nature Limited (2013) and katayama et al. [71] ©American Physical Society (2012).

Light–matter regulation falls within the topic of nonlinear optics in terms of optical science and technology. At lower frequencies, the majority of nonlinear phenomena in the THz regime are optical responses, such as nonlinear transmission, reflection, and absorption in materials driven by strong field THz pulses. Nonlinear THz research differ slightly from those conducted in the optical frequency range [21]. The incident light oscillates with the electric and magnetic degrees of matter in conventional nonlinear optics, imposing forces on the electrons (charge *e*, where *e* is the elementary charge) and the ions (charge *q* = −*e*), thus inducing an electric-dipole density (polarization) ***P***, which is accompanied by a polarized current density d ***P***/d*t*. At the optical wavelength range, oscillations occur at the femtosecond level, where the Coulomb force ***F*** dominates the interaction of light and matter. When the incident light is ***E***, ***F*** can be written as ***F*** = *q* ***E***. As a result, the nonlinear phenomena is predominantly associated with charge distributions (polarization). While THz field oscillations have a significantly longer duration than optical radiation, nonlinear polarization is no longer dominating. Due to the fact that its specific frequencies are capable of driving low-frequency motions such as molecular rotation and crystal lattice vibrations via coupling to ionic, electronic, or spin degrees of freedom (see Figure 2), it can resonantly excite these specific modes in the presence of strong-field THz lightwaves. Furthermore, non-resonant stimulation can cause a variety of nonlinear reactions. When the THz peak field amplitude ***E***_max_ at 1 THz is above 0.3 MV/cm, the ponderomotive energy *W*_*q*_ can reach 1 eV based on the following ponderomotive energy equation $W_{p}=\frac{e^{2}{E_{\max}}^{2}}{4m^{*}\omega^{2}}$ , where m* and *ω* denote effective mass and angular frequency of THz field respectively. This energy is already greater than the ionization energy of an impurity or an excision in a semiconductor, which can result in impact ionization [57–64] and field tunneling effects [39], [40], [41, 59, 65], [66], [67], [68], [69], [70].

However, the absence of cost-effective, highly efficient, polarization-manipulated strong-field THz sources equivalent to those found in other sections of the electromagnetic spectrum is inhibiting the spread of nonlinear THz optics [72–81]. In comparison to ultrashort and super-strong femtosecond laser sources operating at visible and near-infrared frequencies, the single pulse energy, peak field strength, and peak power of ultra-strong THz radiation remain significantly lower. Additionally, several tens of mJ THz pulses with extremely reliable performance are required to develop tiny THz electron accelerators [82]. The significantly greater peak field strength of THz pulses with tunable polarization states and defined temporal structuring has not yet been realized for extreme THz science research. As a result, there is still much space for improvement in the performance of strong-field THz sources. How to achieve leapfrog progress from weak-field passive detection to strong-field triggered treatments also requires extremely intense THz fields. These potentially major uses motivate us to design THz sources that are extremely robust, stable, and user-friendly. Certain questions, such as how to generate more powerful THz radiation, how to increase the efficiency of optical-to-THz energy conversion, and what the strongest THz laser that humans can create, are also extremely interesting scientific and technical challenges.

**Figure 3:**
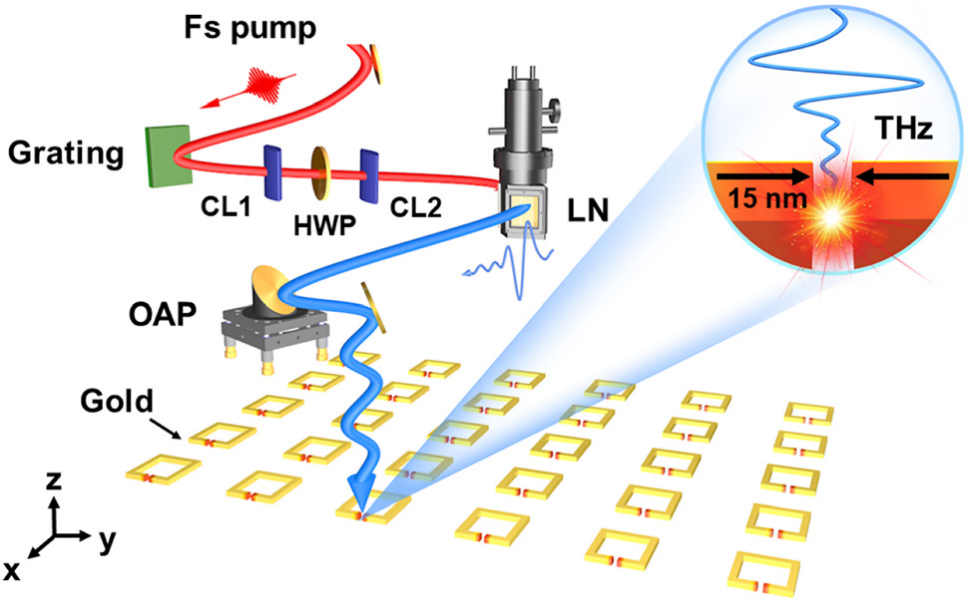
Schematic diagram for achieving high field THz radiation based on lithium niobate or local field enhancement in metasurfaces. CL: cylindrical lens; HWP: half-wave plate; LN: lithium niobate; OAP: off-axis parabolic mirror. Reproduced with permission from Dong et al. [61] ©Wiley-VCH GmbH (2021).

THz frequency, single-pulse energy (photon counts per pulse), energy conversion efficiency, peak electric and magnetic field intensity, and temporal waveform shape are the most important factors in several applications. Apart from typical accelerator-based THz radiation sources, the majority of sources for high field THz in free space are based on femtosecond laser pulse interaction with matter. As demonstrated in Table 1, laser-driven intense THz sources can be classified as photoconductive antennas, plasma in solids, gases, and liquids, and optical rectification [76]. It is noted that THz radiation efficiency is typically calculated by directly dividing the THz single pulse energy by the pumped laser energy applied to the emitting material. However, there is no consensus on the standard for calibrating THz energy probes. As a result, electro-optical (EO) sampling or single-shot diagnosis are used to determine the THz peak field intensity indirectly and thus to correct the detected THz energy. Even so, when ultra-high-frequency THz waves are detected with an EO crystal, linear EO effects frequently exhibit over rotation, whereas nonlinear effects are easily induced by a strong electric field, resulting in measurement error. As a result, novel diagnostic techniques for developing extremely intense THz sources must be investigated. The intuitive option is to generate stronger THz radiation in free space, while the other is to develop a mechanism to realize local field augmentation to match application requirements that are currently unattainable due to the THz radiation’s limiting electric field [57, 60, 61, 83, 84], as illustrated in Figure 3.

**Table 1:** Record numbers for high-field THz sources.

THz sources	Max pulse energy	Efficiency (%)
OR* in LiNbO_3_	1.4 mJ [79]	3.8 [85]
LiNbO_3_ PPLN	0.6 mJ [72]	1.0 [72]
OR* in organic crystal	0.9 mJ [86]	3.0 [86]
OR* in ZnTe	14 μJ [87]	0.7 [87]
Conventional accelerators	600 μJ [88]	n/a
Photoconductive antenna	11 μJ [89]	1.6 [90]
Laser-induced sheath field	0.7 mJ [91]	n/a
Plasma in air and gases	0.19 mJ [73]	2.36 [73]
Laser-solid foil interactions	202 ± 100 mJ [92]	0.29 [92]
Liquids (water)	76 μJ [78]	0.3 [78, 93]

The core concept behind local field enhancement techniques in artificial structures is based on lightning rod and antenna effects. THz fields are confined and localized within metal micro-/nano-gaps, producing resonant or non-resonant field enhancement responses. If artificial structures are delicately created at specific frequencies, they can serve two critical functions. One is used to boost the local field for THz pumping, while the other is used for sensitive probing. This method was widely used in the early years of strong-field THz science and applications, and numerous field-induced impact ionization and tunneling events were identified, culminating in the production of non-equilibrium conductivity in micro-/nano-gaps. This approach allows for localized enhancements of the THz field strength of many orders of magnitude, causing insulator-to-metal phase transitions, nonlinear spin switching, and ultrafast photoluminescence emission, among others. We focus on the lithium niobate crystal-based tilted pulse front approach via optical rectification for generating mJ or even tens of mJ free-space THz radiation in this paper, as well as the creation of nonlinear THz metasurfaces enabled by lithium niobate THz sources.

## 2 Intense THz radiation from lithium niobate

**Figure 4:**
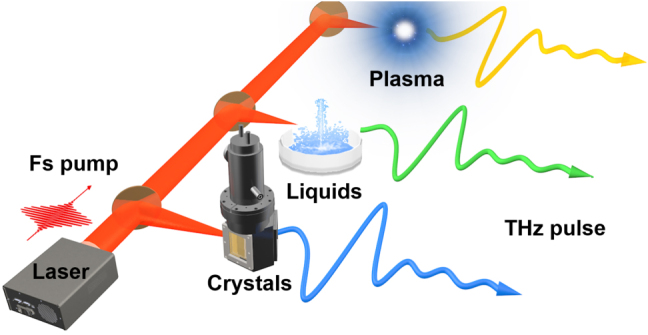
Strong field THz radiation based on nonlinear crystals, liquids, and plasma.

When femtosecond laser pulses interact with solids, liquids, gases, or plasma, strong-field THz radiation can be generated (see Figure 4). Different THz radiation processes exhibit distinct characteristics. While some sources of intense THz radiation are less stable, the emission mechanism is extremely interesting to study. It is not only fascinating in terms of radiation mechanism, but also extremely useful in terms of technology and real-world applications for solid-state THz sources. THz radiation mechanisms in solid-state strong-field sources can be classified as optical rectification (OR), difference frequency generation (DFG), and the inverse spin Hall effect in ferromagnetic metal/heavy metal heterostructures [94–97]. When the radiation single pulse energy, optical-to-THz efficiency, field strength, stability, and expandability are all taken into account, OR and DFG are preferable [86, 98], [99], [100]. OR in lithium niobate for high field THz generation has become ubiquitous in low-frequency applications. What’s more, in 2021, lithium niobate will achieve record levels of single pulse energy with 1.4 mJ, 800 nm-to-THz energy conversion efficiency of 0.7%, and focused electric field strength >6.4 MV/cm.

**Figure 5:**
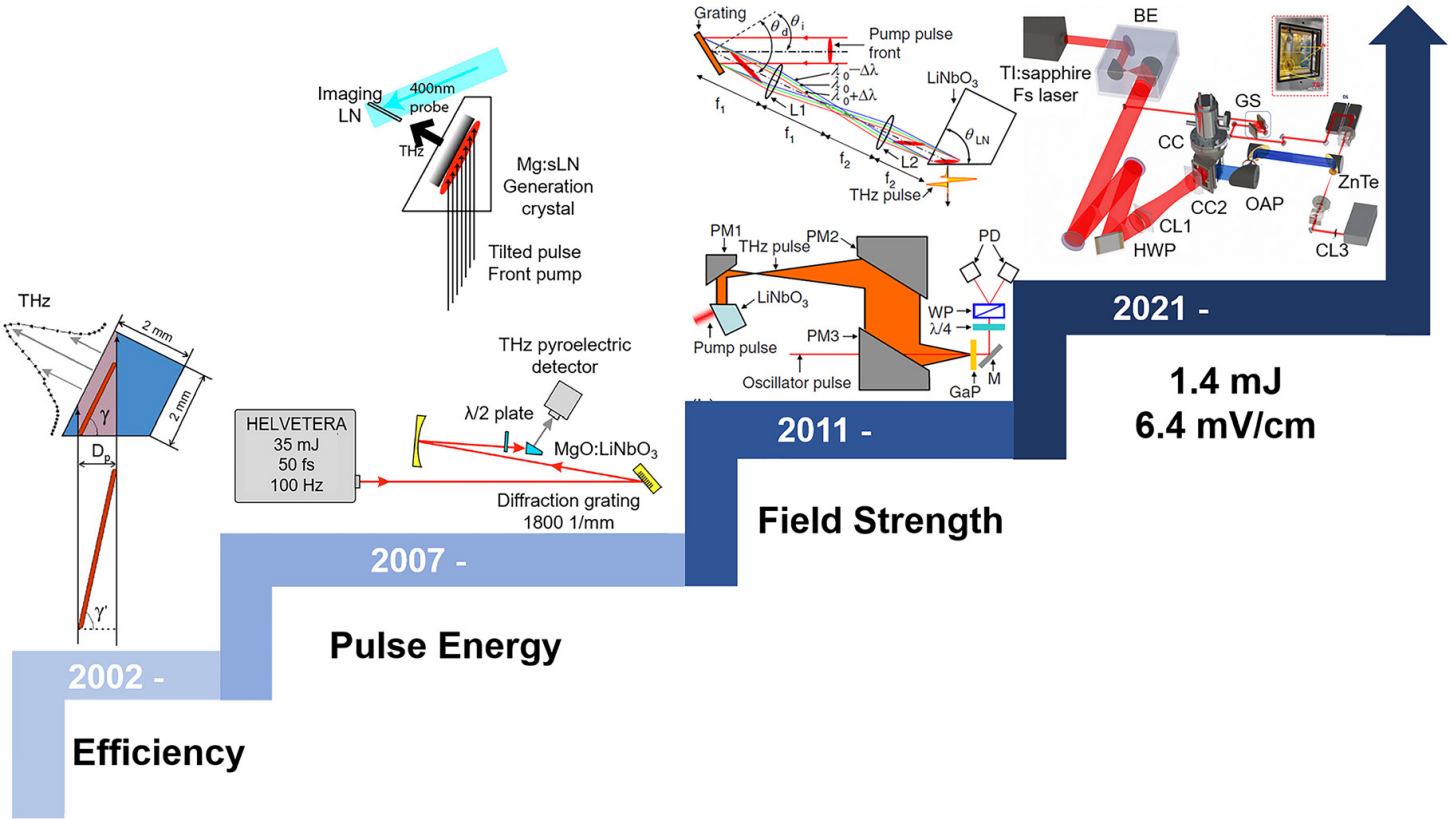
Evolution process for tilted pulse front technique based on lithium niobate. Reprint permission from [102] ©OPTICA (2002) [104]; ©American Institute of Physics (2007) [107]; ©OPTICA (2008) [108]; ©American Institute of Physic (2011) [79]; ©Wiley-VCH GmbH (2021).

Indeed, the first THz pulse was generated from lithium niobate in 1971 [101]. Due to its mature growth technology, lithium niobate can be grown in large sizes and has a high second-order nonlinear coefficient. Additionally, when doped with MgO, lithium niobate crystals can be used without causing damage on Joule-level high power femtosecond lasers. Lithium niobate has a bandgap of 4.0 eV, which allows for effective avoidance of multi-photon absorption during high-energy pumping. However, the phase matching problem and strong linear absorption for THz radiation preclude lithium niobate from being a useful THz source. Fortunately, the tilted pulse front technique was demonstrated successfully in 2002, resolving the phase mismatching problem [102]. Since then, development of strong-field THz sources based on lithium niobate has accelerated. Between 2002 and 2021, the tilted pulse front technique underwent nearly three stages of development, as illustrated in Figure 5. It is roughly equivalent to increasing efficiency, pursuing high single pulse energy, and optimizing focusing for high field strengths [103–106]. THz radiation of greater than 1 mJ has been experimentally demonstrated up to 2021. All efficiency, single pulse energy, and field strength have been increased by more than three orders of magnitude using various optimization techniques. Such tremendous progress is inextricably linked to collaborative experimental and theoretical innovation.

### 2.1 Experimental optimization

As depicted in Figure 3. A typical setup for a tilted pulse front consists of a pumping laser, a grating, an imaging system, and the lithium niobate crystal. As a result, optimization is primarily concerned with the three components. Optimizing pumping laser parameters entails determining the optimal pumping wavelength, pulse duration, spectrum distribution profile, and pumping energy [85, 109], [110], [111], [112], [113]. However, it is difficult to achieve significant improvement solely by optimizing a single parameter. In actual experiments, these parameters are interdependent and frequently require cooperative regulation to produce acceptable experimental results. The primary challenges in optimizing the tilted pulse front optical path are selecting the dispersion component and imaging system. The majority of published results involved the use of gratings to tilt the pulse front of the pumping laser. Additionally, several novel structures have been proposed, including a structure formed by direct etching on the surface of a lithium niobate crystal or contact gratings [114–116]. Furthermore, because the efficiency of diffraction for tilting the pumping pulse fronts is not very high, these novel ideas have not yet gained widespread adoption. The imaging system is the other component. Numerous imaging techniques have been evaluated for increased efficiency, including single convex lenses, double convex lenses, and two cylindrical lenses [117]. Since the successful demonstration of a 1 MV/cm THz field strength with two cylindrical lenses, it has been established that this is the most efficient approach [108]. The nonlinear coefficient for crystal types, absorption for THz waves, and damage threshold are the primary considerations for crystal parameters. Lithium niobate has a relatively high congruent and stoichiometric ratio and has been widely used for strong-field THz generation. However, stoichiometric lithium niobate cannot be grown in large sizes suitable for moderate pumping energy in kHz femtosecond laser systems. Congruent lithium niobate crystals can be grown to extremely large sizes for extremely high power laser excitation. However, its linear absorption of THz waves generated within crystals must be overcome by cryogenically cooling [118]. When the generation crystals are cooled with liquid nitrogen, the efficiency of the radiated THz can be increased [85, 119]. Further cooling the crystal to liquid helium temperature results in a negligible increase in THz efficiency [116]. In terms of damage threshold, MgO doping can enhance the crystal’s performance, allowing for even more J-level femtosecond laser pumping. While mJ-level THz radiation from lithium niobate has been demonstrated successfully, future development will focus on 10-mJ or even Joule-level THz, providing free space THz fields larger than 10 MV/cm or even GV/cm for extreme science and applications. The majority of driving laser systems used in high field THz generation are not purpose-built for it. To generate THz at the Joule level, we must design the pumping laser parameters and the tilted pulse front technique with care, as well as improve theoretical prediction.

### 2.2 Theoretical progress

**Figure 6:**
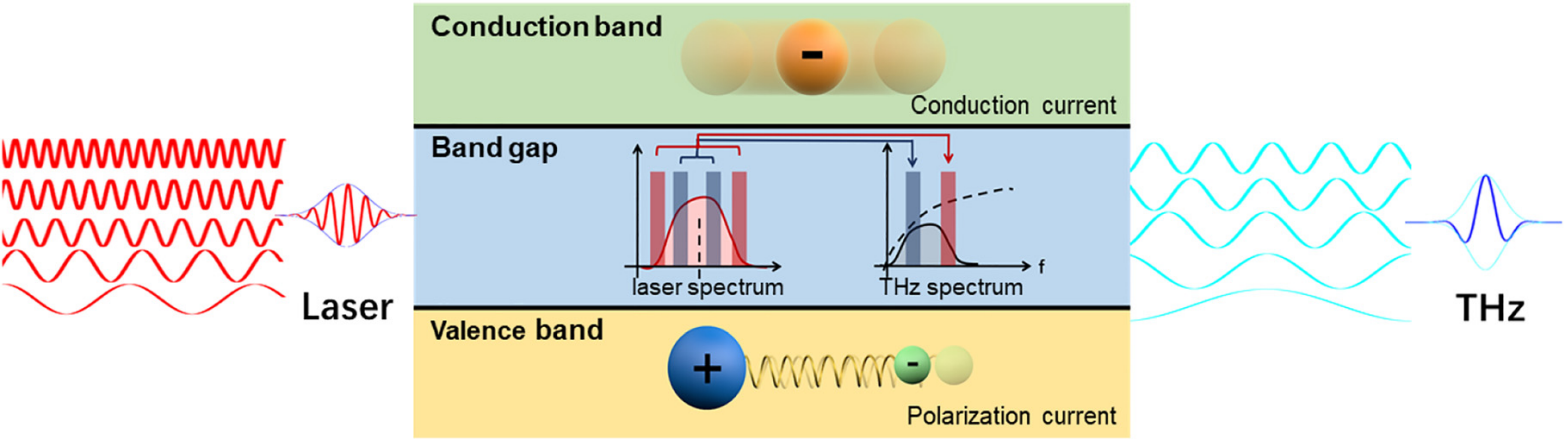
Physical picture of THz radiation from polarization and conduction currents, respectively. Difference frequency generation for THz radiation is inserted in the middle.

The primary goal of tilted pulse front theory is to predict and guide experimental implementation of strong-field THz generation. Its evolution can be roughly classified into four stages: theory proposal, 1D, 2D-3D, and 3D + 1 [120–124]. The theoretical prediction becomes increasingly close to the experimental results as the model is improved continuously. At the outset of the theory proposal, the phase matching wave vector relationship is transformed for the first time into the relationship between the pumping laser’s group velocity and the THz wave’s phase velocity. It illustrates that the two conditions are equal if only considering the first-order angular dispersion and regarding the THz frequency as an infinitesimal quantity compared with the optical frequency. In lithium niobate crystals, the group velocity of laser pulses is naturally larger than the phase velocity of THz waves, which is exactly why collinear phase matching cannot be achieved inside lithium niobate crystals. The titled pulse front technique utilizes the first-order angular dispersion to “decelerate” the group velocity of laser pulses until it synchronized with the phase velocity of THz waves. Consequently, phasing matching condition was satisfied in lithium niobate crystals approximately. It establishes an efficient phase matching scheme in lithium niobate crystals, enabling the experiment to be carried out successfully. The latter researchers derived several models to describe the THz generation process. The main idea of these models is to describe the second-order nonlinear optical processes inside lithium niobate crystals in continuous spectra, including the difference frequency generation of the THz radiation (Figure 6) and the second-order interactions between the THz components and the pump laser pulses. Later, a one-dimensional model was developed that can qualitatively predict the generated THz energy, efficiency, and electric field strength in relation to the pumping laser and crystal parameters. The cascading process has been considered in light of the depletion of the pumping laser spectrum. As a result, the efficiency of optical-to-THz energy conversion should be constrained by the distortion of the pumping laser spectrum within lithium niobate crystals. The efficiency predicted theoretically has been reduced using this method. Then, the above model was extended to two spatial dimensions (2D) to depict the spatio-temporal variations of the optical pump pulses. Further, the 3D+1 numerical model thoroughly investigated the high field THz generation process in the tilted-pulse-front configuration, and it reminded us that the radiated THz pulses and their application experiments must account for significant spatial inhomogeneity. In the future, it will be critical to continue developing the theory for predicting the efficiency and THz properties of lithium niobate via the tilted pulse front technique for Joule-level THz generation. What’s more intriguing is whether the tilted pulse front scheme can be inversely designed to achieve the desired THz characteristics. As a result, a successful combination of experimental and theoretical advances will accelerate the rapid advancement of nonlinear and extreme THz science and applications.

## 3 Nonlinear THz metasurface

Apart from generating free-space strong-field THz radiation for nonlinear THz investigations, another effective method of achieving higher electric fields is by enhancing local fields. By utilizing meta-structures such as metamaterials (metasurfaces), nano-tips, and nano-gaps, among others, one can enhance and localize the incident wave’s electric field. Metamaterials, first proposed in the 1990s [125], are synthetic composites with unit cell sizes typically in the subwavelength range that can manipulate the amplitude, phase, and polarization of incident electromagnetic waves. Metasurfaces are a two-dimensional representation of their three-dimensional form, which is more easily realized and fabricated. The most attractive property of nonlinear THz metasurfaces is their strong ability to enhance and localize fields. Since the first experimental realization of a THz-induced vanadium dioxide phase transition in 2012 [84], nonlinear THz investigations have been strongly correlated with metasurfaces, providing an extremely effective scheme for studying nonlinear THz phenomena with a moderate free-space THz field strength of kV/cm. We review the development of the nonlinear THz metasurface in this section, with an emphasis on electric field enhancement.

### 3.1 Resonant-enhanced nonlinear THz metasurface

**Figure 7:**
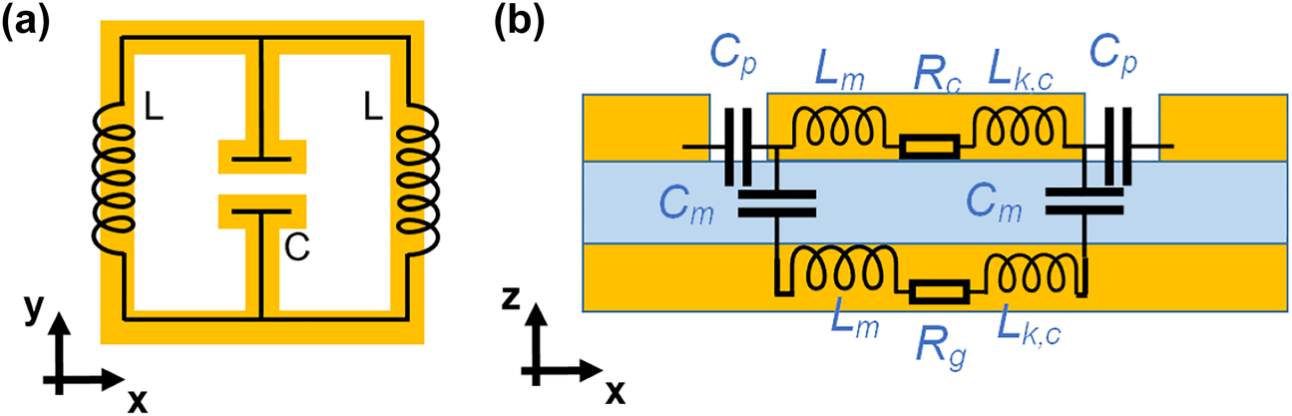
Classical lumped equivalent-circuit model for (a) split-ring resonators and (b) perfect absorber metasurfaces.

As previously stated, a split-ring resonator (SRR) metasurface (two-dimensional metamaterial) can be used to resonantly enhance picosecond and high-field THz pulses, thereby lowering the coulomb-induced potential barrier for carrier transport. The enhancement of the electric field is due to the inductance-capacitance (LC) resonance, which can be modeled using lumped equivalent circuits [126, 127]. Numerous metasurface phenomena can be adequately explained using this model, and numerous related devices are demonstrated. As illustrated in Figure 7, the gap and spacer in both SRRs [57, 59, 64, 84, 128] and perfect absorbers [60] can be viewed as capacitance, while the metal structure can be viewed as inductance and resistance. At resonant frequencies, the E field is amplified and localized at the gap, inducing an intensity-dependent response in the matter. Additionally, according to theory, the dielectric environments surrounding micro-/nano-gaps vary, which can be detected sensitively using a coherent THz probing beam.

**Figure 8:**
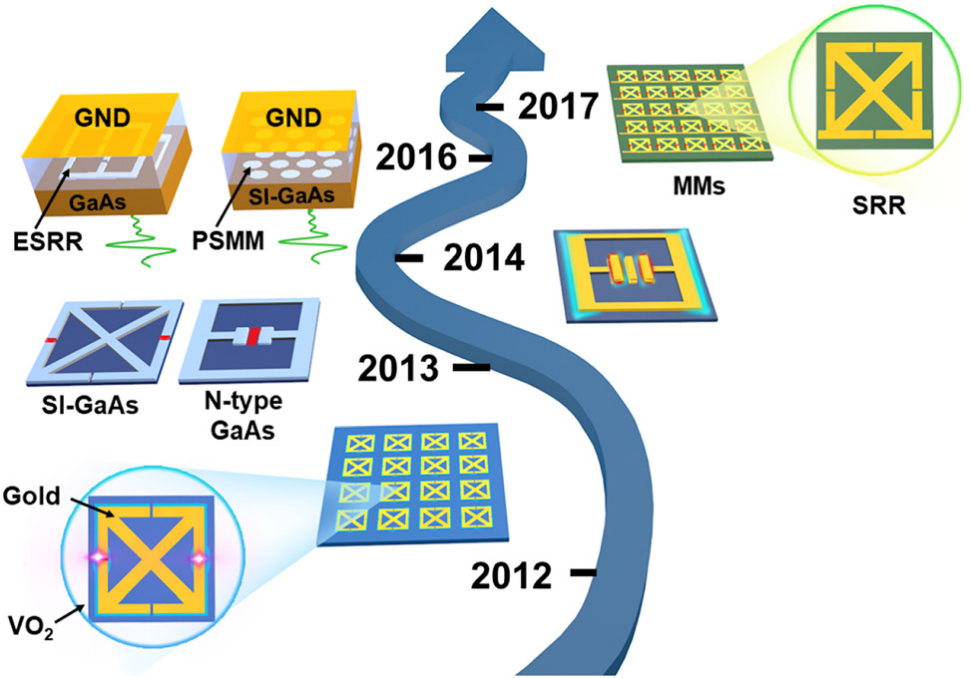
Timeline of the development on THz split-ring resonator nonlinear metasurfaces. Reproduced with permission from [21] ©Springer Nature Limited (2013) [57, 59]; ©American Physical Society (2014) [60]; ©OPTICA (2016) [129]; ©American Institute of Physics (2017).

The research on resonance-enhanced nonlinear THz metasurfaces is primarily concerned with structural engineering in order to increase the strength of the local THz field and then induce nonlinear responses in various substrate materials. The majority of reported investigations into meta-structure design and fabrication utilized micro-gap-enabled SRRs. These structures multiply the local field enhancement by several tens, which is not possible with traditional lithography. Nonetheless, such enhancements for free-space THz fields of several hundred kV/cm can ensure that local fields exceed MV/cm. As a result, numerous nonlinear responses have been observed experimentally in phase transition materials and semiconductors [57, 59, 61, 63, 84]. The nonlinear behavior of phase transition materials such as VO_2_ film [84] is due to the insulator to metal transformation caused by strong field THz excitation. However, the mechanism of local field-induced nonlinearity is more complex in semiconductors, and can be classified as impact ionization [57, 59, 61, 63], intervalley scattering [57, 61], and interband tunneling [59]. To disentangle their contributions to THz nonlinearity, the doping level, incident field strengths, and substrate material types were all explored in detail. In 2013, the SRR metasurface (gap ∼ 2.2 μm width) was deposited on 1.8 μm-thick doped (1 × 10^16^ cm^−3^) GaAs films. Intervalley scattering was observed at free-space THz peak fields of ∼20–160 kV/cm [57], which can reduce carrier mobility and enhance the SRR response due to a conductivity decrease in the doped thin film. When the THz field was greater than 160 kV/cm, the increased electric field resulted in impact ionization, which increased carrier density and conductivity. Intriguingly, in 2014, SRR metamaterials (gap ∼ 2.5 μm width) were fabricated on an undoped GaAs substrate, where the THz field strength can reach the ponderomotive energy in the keV range. Non-destructive quasistatic interband tunneling and impact ionization were demonstrated experimentally by observing ultra-broadband near-infrared and visible interband luminescence induced by the enhanced THz local field with ∼10 MV/cm [59], as illustrated in Figure 8. Except these non-tunable resonantly enhanced nonlinear THz metasurfaces, an electrically active nonlinear THz SRR metamaterial (∼1 μm width) was proposed [129]. External DC bias can be used to control the resonance’s strength and nonlinearity, resulting in the nonlinear THz metasurface functionalization. In short, nonlinear THz phenomena can be investigated without being completely constrained by the free-space THz field strength using resonant metasurfaces with micrometer gaps.

### 3.2 Non-resonant nonlinear THz metasurfaces

**Figure 9:**
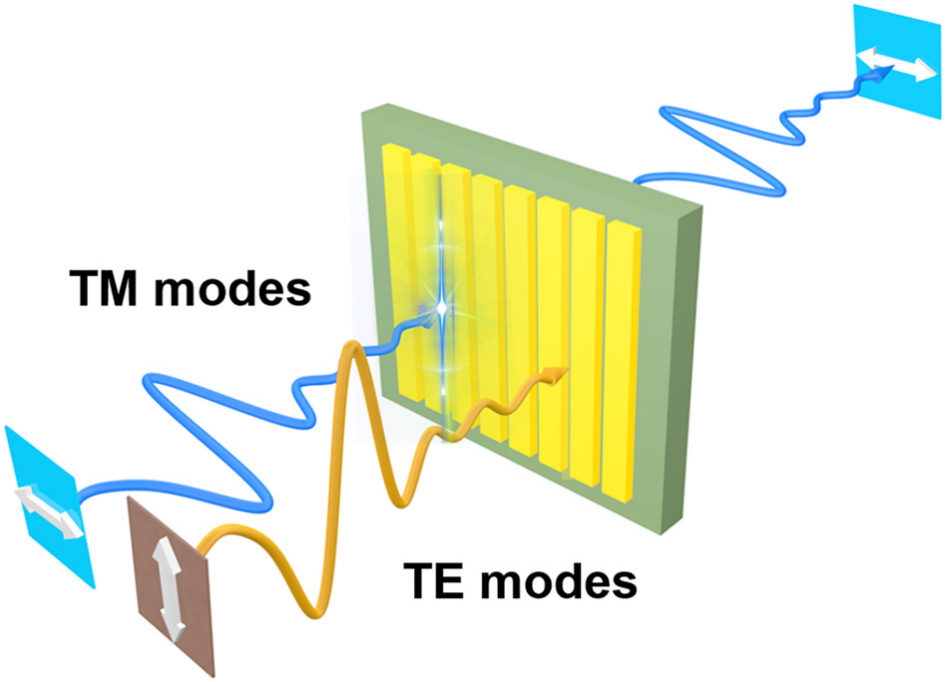
The schematic of the nano-slot metasurfaces (one-dimensional subwavelength grating).

Resonant metasurfaces have been proved to be an effective way to enhance the THz electric field. However, the resonance can only enhance the localized field for certain frequencies. For broadband high field THz pulses, non-resonant metasurfaces are proposed for general local field enhancement. Normally, such kinds of structures, as illustrated in Figure 9, are constructed by one-dimensional subwavelength grating or nano-slot metasurfaces [65, 68, 70, 130], Metasurfaces with resonant properties have been shown to be an effective method of increasing the THz electric field. However, resonance can enhance the localized field only for a limited range of frequencies. Non-resonant metasurfaces are proposed for general local field enhancement with broadband high field THz pulses. Typically, such structures are constructed using one-dimensional subwavelength gratings or nano-slot metasurfaces that support transmitting TM mode but not TE mode. As a result, the only way for THz waves to transmit through this grating is through the gaps or slots. Local field enhancement is not frequency dependent in this case, but is highly dependent on the gap size and duty ratio.

**Figure 10:**
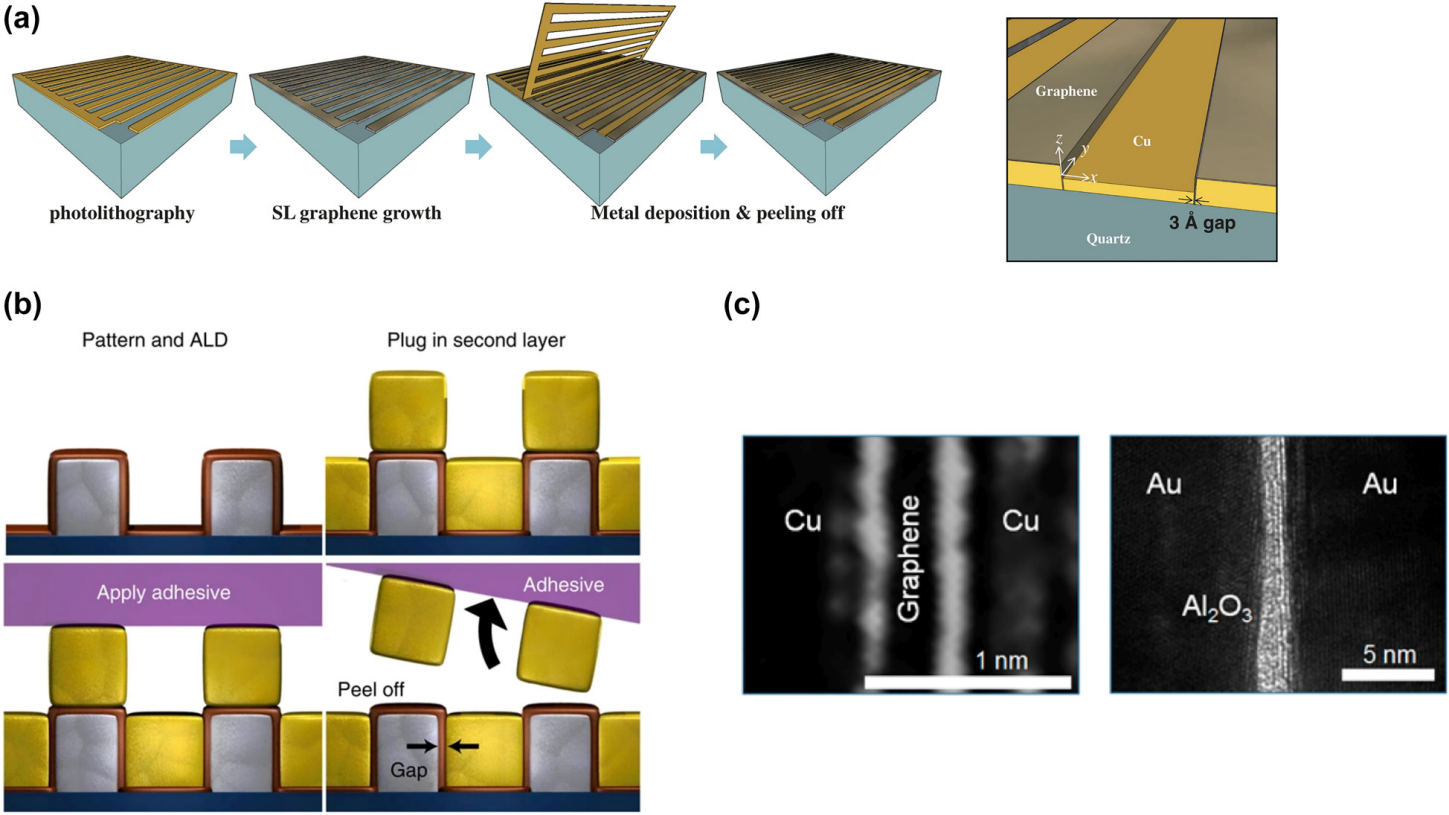
Nonlinear THz nano-slots based on “plug-and-peel” metal patterning technique for observing nonlinear phenomena. (a) Graphene [130], (b) Al_2_O_3_ [68, 131]. (c) SEM images of the metasurfaces. Reproduced with permission from [130] ©American Physical Society (2015) [68]; ©American Chemistry Society (2015) [131]; ©Springer Nature Limited (2013).

As a result, for the gap parameters, micro-, nano-, and even angstrom-scale values can be used to achieve extremely effective local field enhancement. More intriguingly, a fabrication technique called “plug-and-peel” metal patterning [68, 130] has been developed to enable the creation of slots of varying sizes. In Figure 10, a graphene atomic layer is shown acting as an angstrom-sized plug. When it is peeled away, nano-slots between the metal stripes form, resulting in metal-nano-spacer-metal metasurfaces [130]. The slot width can be reduced to 0.3 nm using this method, resulting in a 5 × 10^7^ times. fold increase in the localized field enhancement factor. Due to the extremely large ponderomotive energy, such high fields can induce electron tunneling. The presence of obvious THz nonlinearity is detected. Additionally, insulators such as Al_2_O_3_ are used to replace graphene, and numerous nonlinear responses in the THz regime are observed.

**Figure 11:**
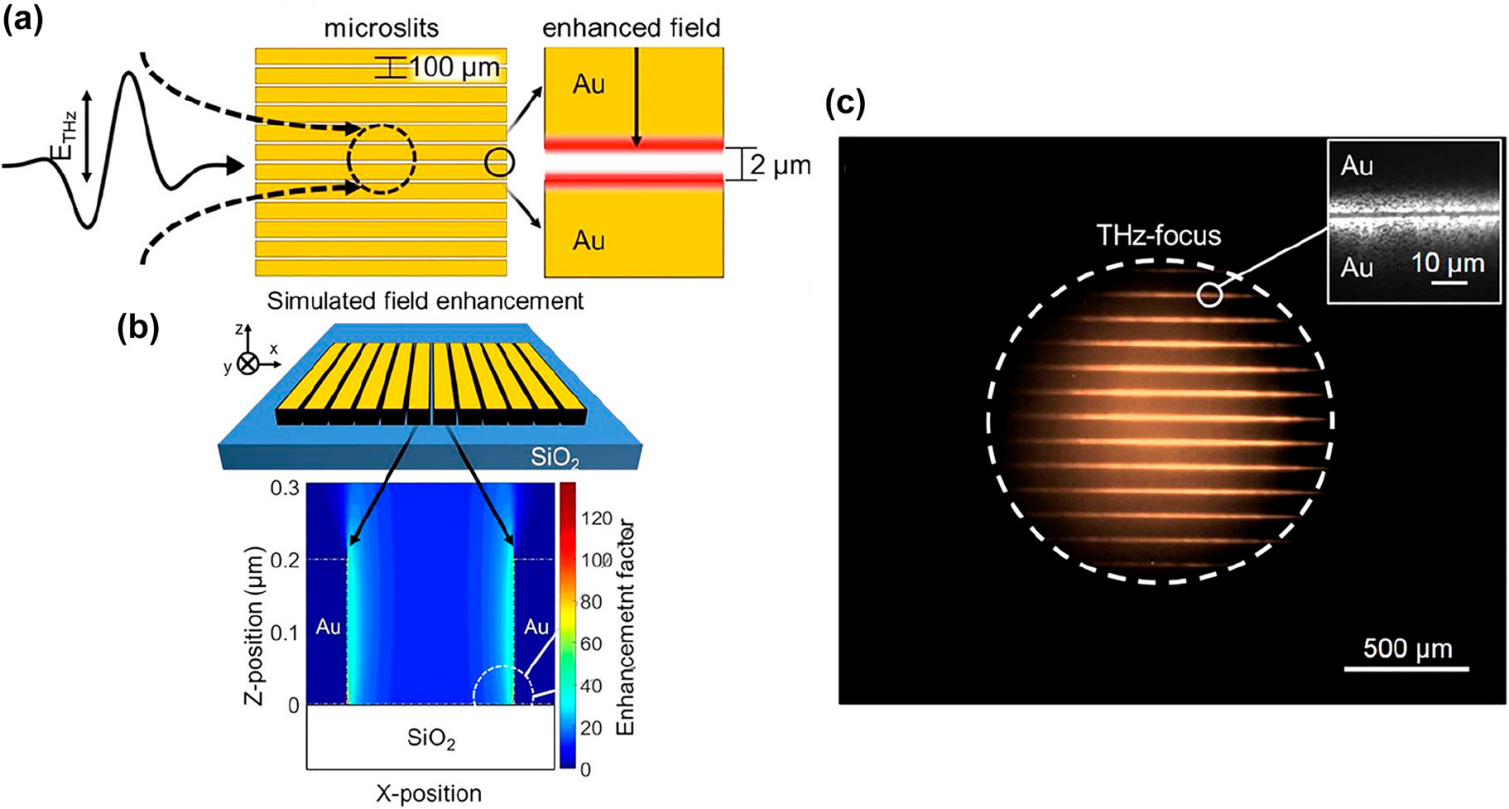
Strong field THz induced quantum dots luminescence [70]. (a) The THz field was enhanced in the 2 μm capacitive gaps between the gold lines, where quantum dots were deposited over; (b) simulation of microslit THz near-field enhancement; (c) focusing THz pulses onto samples generates a visible light image. Reproduced with permission from Pein et al. [70] ©American Chemistry Society (2017).

Similarly, metal nano-slots have been used to enhance the THz field and further stimulate the luminescence of CdSe–CdS core–shell quantum dots [70]. When the THz driving field is increased to 100 kV/cm, the quantum dots begin to glow and become visible to the naked eye (Figure 11). The ponderomotive energy is calculated to be greater than 0.5 eV in this case, with an unusually high and rapid modulation of the bandgap.

**Figure 12:**
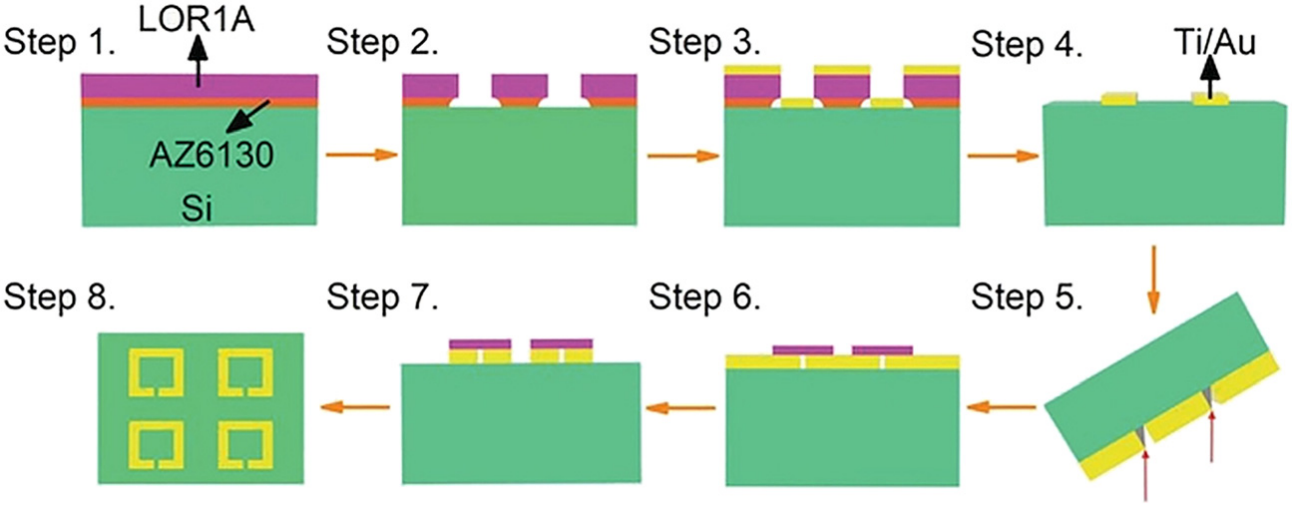
Fabrication technique for large-scale THz metasurface with nano-gap. Reproduced with permission from Dong et al. [61] ©Wiley-VCH GmbH (2021).

In short, non-resonant metasurfaces can also effectively enhance the electric field, and numerous nonlinear THz responses have been achieved as a result of the high ponderomotive energy. Particularly when the gap is on the nanometer scale, the electric field can be magnified by a factor of five. Recalling what we discussed previously about resonant metasurfaces, an intriguing question is whether these two types of enhancement structures can be effectively combined to investigate THz nonlinearity. The majority of gap widths in resonant metasurfaces are greater than 1 μm, and the localized (in-gap) electric field enhancement is typically less than 50 times. The primary difficulty in combining resonant and non-resonant metasurfaces is narrowing the gap size in resonant metastructures with large sample sizes to the nanometer scale. To accomplish this, a THz SRR metasurface with a nano-gap [61] is created by combining traditional lithography and a novel procedure (Figure 12).

**Figure 13:**
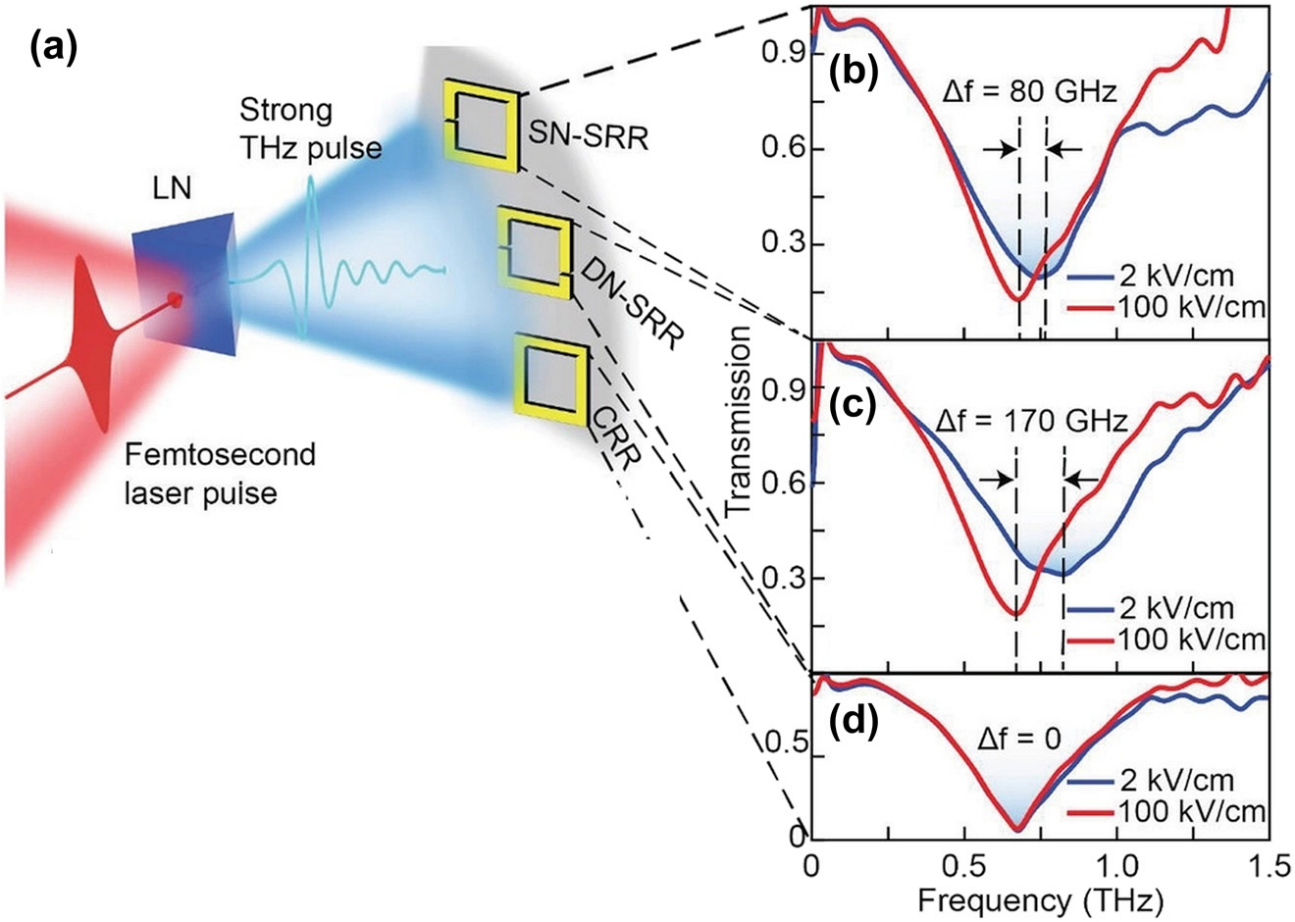
Nonlinear THz response for the THz SRR metasurface with nano-gaps. (a) The strong field THz pulse is generated via titled wavefront technology in a lithium niobate crystal, and then is illuminated onto three different types of metasurfaces. Measured normalized transmitted spectra for (b) SN-SRRs, (c) DN-SRRs, and (d) CRRs under 2 and 100 kV/cm THz illuminations. Reproduced with permission from Dong et al. [61] ©Wiley-VCH GmbH (2021).

Using this nano-gap SRR metasurface, nonlinear THz phenomena on silicon substrate are observed.When the THz electric field is increased from 2 to 100 kV/cm for single nano-gap SRRs, the resonance frequency is redshifted by 80 GHz due to field-induced carrier multiplication in the silicon substrate in the nano-gaps, as illustrated in Figure 13. When the capacity cascade effect is taken into account and the nano-gap numbers are increased, the nonlinear effect can even be enlarged relatively. Additionally, via inter-valley scattering and auger recombination, strong-field THz can reduce the density of photocarriers injected by the optical pump. As a result, the approach advances to a new level by enabling the fabrication of enhanced nonlinear nano-/micro-composites for field-sensitive extreme THz nonlinear applications without the use of intense THz light sources.

In summary, the metal metasurface can be used to localize high fields, and the nonlinear phenomena are caused by transient material changes induced by the THz *E*-field. Both nonlinear mechanisms can contribute to ponderomotive energy, a non-resonant material regulation property, in both resonant and non-resonant metasurfaces. The resonant THz metasurface has not been implicated in the regulation of resonant materials, including coupling with molecular rotation and crystal lattice vibrations. Additionally, it was demonstrated that hot Dirac fermions can generate THz harmonics in graphene with high efficiency [132]. A few theoretical and experimental studies demonstrate that using graphene metasurfaces significantly improves THz harmonic generation [133, 134], and it is expected that experiments with topological surface states, such as Dirac and Weyl semi-metals [5, 135], will be reported. Zhao et al. demonstrated unequivocally that metasurfaces have advantages in nonlinear optics [136] and that these techniques could also be applied to nonlinear THz phenomena in topological surfaces. Metal metasurfaces can be deposited directly on Dirac or Weyl semi-metal surfaces to achieve THz nonlinearity via the metasurfaces’ resonance localization effect [137]. Moreover, nonlinearity may be achieved by using strong THz pumping to the patterned Dirac or Weyl semi-metal material [137–139], viewed as the metasurface structure, such as optical bound states in continuum. Metasurfaces, we anticipate, will allow for the realization of more novel nonlinear THz phenomena.

## 4 Perspective

**Figure 14:**
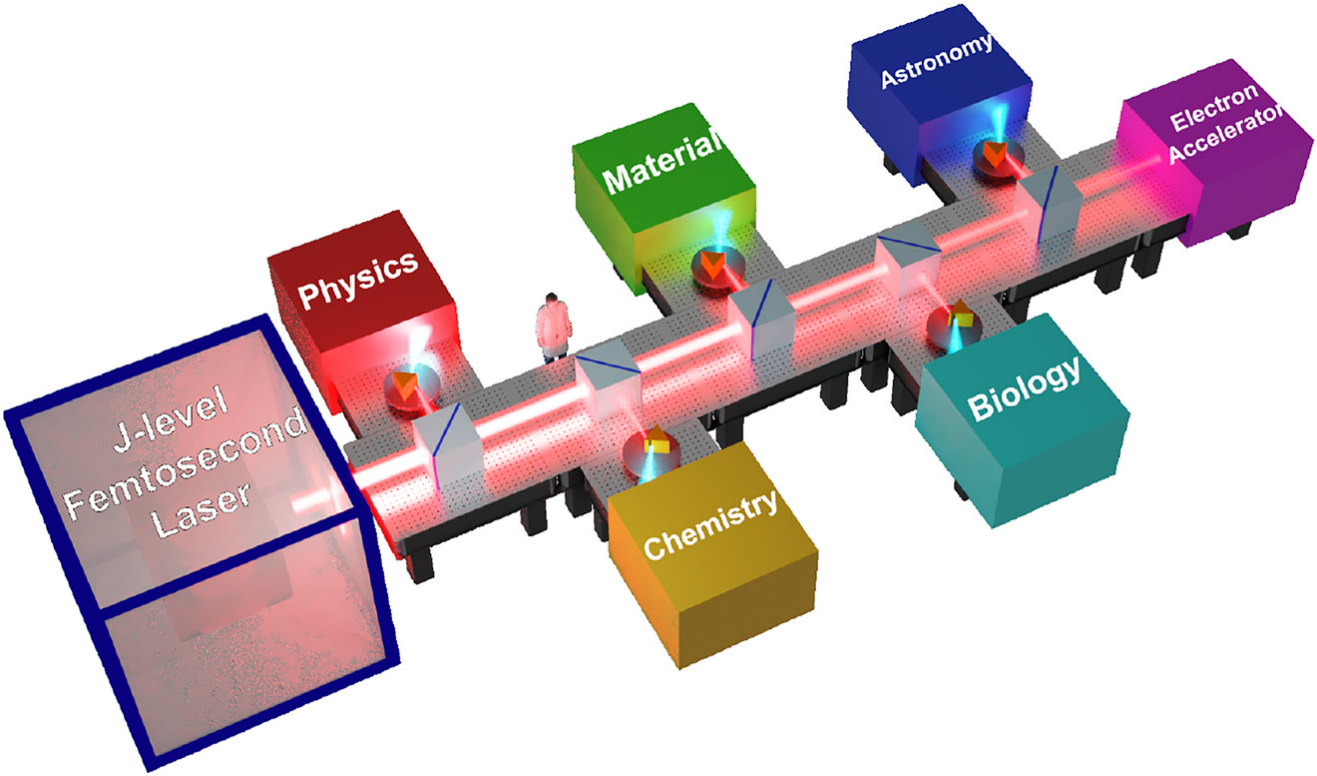
Intense THz sources enabled interdisciplinary applications.

The field strength of the international advanced THz light source is currently on the order of MV/cm, which is comparable to the electric field strength between semiconductor atoms and induces non-equilibrium states via electromagnetic field action and driving phonon coupling. If the field strength is increased further to GV/cm (∼300 Tesla), it will be able to match the interatomic electric field in metals (∼100 MV/cm), and thus will be capable of inducing more extreme non-equilibrium states. By increasing the energy of a single pulse, implementing ultra-broadband emission, increasing the peak frequency, and shrinking the light spot, it is possible to achieve an electric field intensity of GV/cm, while nano-gap or nano-metal tip field enhancement may also be a method. In a strong field, the nonlinear effect is expected to realize multi-body coupling and novel quantum states. Additionally, to meet the demand for material regulation, next-generation super-strong THz light sources must improve their pulse energy, field strength, polarization, and frequency tunability, which can be accomplished through the development of advanced THz source technology and the construction of more versatile THz metasurfaces. The integration of disciplines will significantly accelerate the development of extreme THz science and applications, and will truly benefit fundamental scientific research and potential applications in the future (Figure 14).

**Figure 15:**
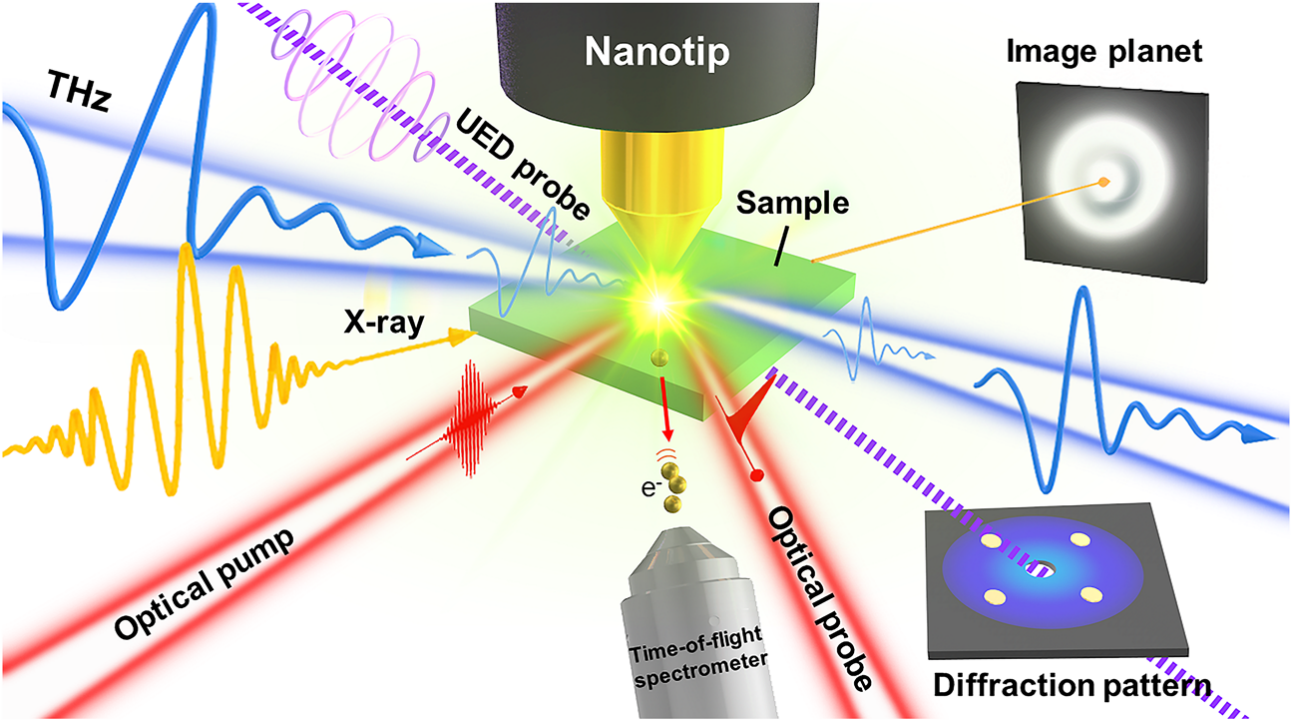
Strong-field THz coupled optical, UED, or X-ray probing system with nanophotonics methods.

THz science and technology have advanced to unprecedented levels, demonstrating that THz electromagnetic radiation has evolved into a versatile frequency band for the discovery of novel physics and phenomena, as well as a powerful tool for scientific frontier research, particularly when coupled with other advanced measurement technologies in interdisciplinary research, such as THz pump-THz/optical/X-ray/ultrafast electron diffraction probe (see Figure 15).
